# 地舒单抗与唑来膦酸治疗新诊断多发性骨髓瘤骨病的疗效及安全性

**DOI:** 10.3760/cma.j.cn121090-20231203-00289

**Published:** 2024-04

**Authors:** 艺 马, 秀斌 肖, 耀升 刘, 喜林 陈, 顺宗 袁, 世华 赵, 云 鲁, 华 殷, 俊丽 陈, 玥琦 王, 娜娜 程, 盼 封, 文荣 黄

**Affiliations:** 1 解放军总医院第五医学中心血液病医学部淋巴瘤-浆细胞疾病专科，北京 100071 Department of Lymphoma & Plasma Cell Disease, Senior Department of Hematology, the Fifth Medical Center of PLA General Hospital, Beijing 100071, China; 2 解放军总医院第四医学中心骨科医学部，北京 100048 Senior Department of Orthopedic, the Fourth Medical Center of PLA General Hospital, Beijing 100048, China

**Keywords:** 多发性骨髓瘤, 骨疾病, 低钙血症, 地舒单抗, Multiple myeloma, Bone disease, Hypocalcemia, Denosumab

## Abstract

**目的:**

探讨地舒单抗（DENOS）与唑来膦酸（ZOL）治疗新诊断多发性骨髓瘤骨病（MBD）的疗效及安全性。

**方法:**

回顾性分析2021年3月1日至2023年6月30日解放军总医院第五医学中心血液病医学部收治的80例新诊断MBD患者的临床资料。18例伴重度肾损害（SRI）患者［内生肌酐清除率（CrCl）<30 ml/min］均接受DENOS治疗，62例非SRI患者分为DENOS组（30例）和ZOL组（32例）。

**结果:**

80例MBD患者中26例（33％）发生低钙血症，22例发生于第1次用药后。非SRI患者中DENOS组低钙血症发生率高于ZOL组［20％（6/30）对13％（4/32），*P*＝0.028］，SRI患者低钙血症发生率为89％（16/18）。多因素分析显示，CrCl<30 ml/min与DENOS治疗后低钙血症相关（*P*<0.001）。抗骨吸收药物治疗1个月后，DENOS组SRI、非SRI患者血清Ⅰ型胶原交联羧基端肽β特殊序列降低率大于ZOL组（68％对59％对27％，*P*<0.001），DENOS组SRI、非SRI患者血清Ⅰ型原胶原氨基端前肽升高率大于ZOL组（34％对20％对11％，*P*<0.05）。抗骨吸收药物治疗后各组全段甲状旁腺激素升高。所有患者均未发生抗骨吸收药物相关颌骨坏死及肾脏不良事件，各组血液学总有效率、完全缓解率、严格意义的完全缓解率差异均无统计学意义（*P*值均>0.05），中位无进展生存及总生存时间均未达到。

**结论:**

DENOS治疗MBD具有较强的抗骨吸收作用和低肾毒性，低钙血症是常见不良反应，多为轻中度且可控。

## Abstract (en)

**Objective:**

This study investigated the efficacy and safety of denosumab（DENOS）versus zoledronic acid（ZOL）in the bone disease treatment of newly diagnosed multiple myeloma.

**Methods:**

The clinical data of 80 patients with myeloma bone disease（MBD）at the Fifth Medical Center of PLA General Hospital between March 1, 2021 and June 30, 2023 were retrospectively reviewed. Eighteen patients with severe renal impairment（SRI, endogenous creatinine clearance rate<30 ml/min）were treated with DENOS, and 62 non-SRI patients were divided into DENOS（30 patients）and ZOL group（32 patients）.

**Results:**

Hypocalcemia was observed in 26（33％）patients, and 22 patients developed hypocalcemia during the first treatment course. The incidence of hypocalcemia in the non-SRI patients of DENOS group was higher than that in the ZOL group［20％（6/30）*vs* 13％（4/32）, *P*＝0.028］. The incidence of hypocalcemia in SRI was 89％（16/18）. Multivariate logistic regression analysis revealed that endogenous creatinine clearance rate <30 ml/min was significantly associated with hypocalcemia after DENOS administration（*P*<0.001）. After 1 month of antiresorptive（AR）drug application, the decrease in the serum β-C-terminal cross-linked carboxy-telopeptide of collagen type I concentrations of SRI and non-SRI patients in the DENOS group were significantly higher than that in the ZOL group（68％ *vs* 59％ *vs* 27％, *P*<0.001）. The increase in serum procollagen type Ⅰ N-terminal propeptide concentrations of patients with or without SRI in the DENOS group were significantly higher than that in the ZOL group（34％ *vs* 20％ *vs* 11％, *P*<0.05）. The level of intact parathyroid hormone in each group increased after AR drug treatment. None of the patients developed osteonecrosis of the jaw and renal adverse events, and no statistically significant differences in the overall response rate, complete remission and stringent complete remission rates were found among the groups（*P*>0.05）, and the median PFS and OS time were not reached（*P*>0.05）.

**Conclusion:**

In the treatment of MBD, DENOS minimizes nephrotoxicity and has strong AR effect. Hypocalcemia is a common adverse event but is usually mild or moderate and manageable.

多发性骨髓瘤（MM）是一类恶性浆细胞疾病，约80％新诊断MM（NDMM）伴溶骨性破坏[1]，易发生骨相关事件（SREs），包括病理性骨折、脊髓压迫，必要时需骨科手术或放疗干预，SREs使患者的生活质量和生存受到严重影响[2]。双膦酸盐（BPs）是治疗骨髓瘤骨病（MBD）的传统药物，但随着对MBD病理生理机制的深入了解，首个靶向核因子κB受体活化因子配体（RANKL）的单克隆抗体地舒单抗（denosumab, DENOS）应用于临床。有前瞻性研究证实，DENOS对于延迟首次SREs发生时间不劣于第三代BPs唑来膦酸（zoledronic acid, ZOL）[3]，且DENOS不经肾脏代谢的特点满足了伴重度肾损害（SRI）MM患者的需求[4]。解放军总医院第五医学中心血液病医学部淋巴瘤-浆细胞疾病专科回顾性分析了DENOS与ZOL治疗新诊断MBD期间骨转换标志物（BTMs）及血钙变化，初步探讨其疗效及安全性。

## 病例与方法

1. 病例：回顾性分析2021年3月1日至2023年6月30日解放军总医院第五医学中心血液病医学部淋巴瘤-浆细胞疾病专科收治的80例伴溶骨性破坏NDMM患者的临床资料。所有患者经影像学检查结果（CT、MRI或PET-CT）证实存在至少一处溶骨性破坏病灶（≥5 mm），MBD诊断标准依据IMWG标准[5]。根据影像学检查结果对MBD进行分级[6]：0级为无骨病变；1级为弥漫性骨质疏松；2级为1个解剖学部位的1个或多个溶骨性破坏；3级为多个解剖学部位的多个溶骨性破坏；4级为严重溶骨性破坏并发病理性骨折。

2. 治疗方案：患者接受的一线诱导方案见表1。适合行自体造血干细胞移植（auto-HSCT）者诱导治疗4～6个周期后序贯auto-HSCT，不适合行auto-HSCT者诱导治疗9～12个周期，28 d为1个周期。维持治疗采用来那度胺或来那度胺联合硼替佐米。治疗期间口服阿昔洛韦预防带状疱疹病毒激活，阿司匹林预防来那度胺相关静脉血栓。

**表1:** 80例多发性骨髓瘤骨病患者的临床资料

组别	地舒单抗组		唑来膦酸组（32例）	*P*值^a^	总体（80例）
	CrCl<30 ml/min（18例）	CrCl≥30 ml/min（30例）			
男性[例（%）]	11（61）	18（60）	20（63）	0.840	49（61）
年龄[岁，*M*（范围）]	56（44～90）	58（41～86）	59（44～84）	0.693	59（41～90）
免疫球蛋白类型[例（%）]				0.975	
IgG	10（55）	17（57）	19（59）		46（57）
IgA	3（17）	6（20）	7（22）		16（20）
IgD	1（6）	1（3）	1（3）		3（4）
轻链	4（22）	6（20）	5（16）		15（19）
ISS分期Ⅲ期[例（%）]	17（94）	13（43）	14（44）	0.974	44（55）
DS分期Ⅲ期[例（%）]	15（83）	25（83）	27（84）	1.000	67（84）
ECOG评分[例（%）]				0.830	
<3分	15（83）	25（83）	26（81）		66（82）
≥3分	3（17）	5（17）	6（19）		14（18）
MBD分级[例（%）]				0.966	
2级	4（22）	6（20）	6（19）		16（20）
3级	3（17）	10（33）	10（31）		23（29）
4级	11（61）	14（47）	16（50）		41（51）
细胞遗传学高危[例（%）]	10（56）	12（40）	13（41）	0.960	35（44）
CrCl[ml/min，*M*（范围）]	16（6～29）	98（40～162）	95（35～148）	0.662	78（6～162）
高钙血症[例（%）]	6（33）	3（10）	3（9）	1.000	12（15）
SREs[例（%）]				0.997	
病理性骨折	11（61）	14（47）	16（50）		41（51）
脊髓压迫	1（6）	3（10）	4（13）		8（10）
骨科手术	3（17）	11（37）	13（41）		27（34）
骨放疗	0（0）	1（3）	1（3）		2（3）
诱导治疗方案[例（%）]				0.763	
VCd	5（28）	3（10）	3（9）		11（13）
VRd	0（0）	23（77）	24（75）		47（59）
VPd	8（44）	0（0）	0（0）		8（10）
Vd-PACE	0（0）	3（10）	2（6）		5（6）
Dara-VCd	3（17）	1（3）	3（9）		7（9）
SVPd	2（11）	0（0）	0（0）		2（3）
自体造血干细胞移植[例（%）]	10（56）	17（57）	17（53）	0.779	44（55）
AR药物应用次数[次，*M*（范围）]	16（5～22）	17（6～24）	17（6～23）	0.849	17（5～24）
AR药物暴露时间[月，*M*（范围）]	18（5～24）	18（6～24）	18（6～24）	0.656	18（5～24）

**注** ^a^*P*值：地舒单抗组CrCl≥30 ml/min患者与唑来膦酸组比较；ISS：国际分期体系；DS：Durie-Salmon分期体系；ECOG：美国东部肿瘤协作组；MBD：骨髓瘤骨病；细胞遗传学高危：FISH检测出t（4；14）、t（14；16）、t（14；20）、del（17p）、1q21扩增；CrCl：内生肌酐清除率；SREs：骨相关事件；VCd：硼替佐米+环磷酰胺+地塞米松；VRd：硼替佐米+来那度胺+地塞米松；VPd：硼替佐米+泊马度胺+地塞米松；Vd-PACE：硼替佐米+地塞米松+卡铂+表柔比星+环磷酰胺+依托泊苷；Dara-VCd：达雷妥尤单抗+硼替佐米+环磷酰胺+地塞米松；SVPd：塞利尼索+硼替佐米+泊马度胺+地塞米松；AR：抗骨吸收

ZOL 4 mg静脉滴注至少15 min，内生肌酐清除率（CrCl）在30～60 ml/min时需调整ZOL剂量，且禁用于伴SRI（CrCl<30 ml/min）患者；DENOS 120 mg，皮下注射。SRI患者接受DENOS治疗。ZOL和DENOS均为每4周给药1次，计划持续2年，治疗期间每日补钙（≥500 mg）及维生素D（≥400 IU），高钙血症患者待血钙降至正常后开始。

3. 口腔卫生管理：治疗期间每6个月进行1次口腔常规检查，或出现口腔不适时就诊。保持良好的口腔卫生，使用牙科器械或拔牙时需停用ZOL 6个月（操作前后各停药3个月）[7]；或提前1个月停用DENOS，待手术创面愈合后继续用药[8]。

4. BTMs及血钙检测：BTMs检测时间点为治疗前和治疗后1、2、3、4、5个月。采用电化学发光法检测血清BTMs，包括Ⅰ型胶原交联羧基端肽β特殊序列（β-CTX）、Ⅰ型原胶原氨基端前肽（PINP）、全段甲状旁腺激素（iPTH）。入组患者均接受血清钙检测，校正血清钙计算公式：校正血清钙（mmol/L）＝血清钙（mmol/L）−0.025×血清白蛋白浓度（g/L）+1.0（mmol/L）。校正血清钙<2.10 mmol/L为低钙血症，≥2.75 mmol/L为高钙血症。

5. 血液学疗效及安全性评估标准：血液学疗效评价采用IMWG标准[5]，分为严格意义完全缓解（sCR）、完全缓解（CR）、非常好的部分缓解（VGPR）、部分缓解（PR）、微小缓解（MR）、疾病稳定（SD）、疾病进展（PD）。安全性评价依照美国常见不良反应术语评定标准5.0版分级标准评定。

6. 随访：随访截止日期为2023年11月20日，无进展生存（PFS）期定义为自诊断之日起至任何原因导致疾病进展或死亡的时间，总生存（OS）期定义为自诊断之日起至任何原因导致死亡的时间。

7. 统计学处理：采用Graphpad Prism 9.5统计学软件进行数据分析，计数资料以例数（％）表示，符合正态分布的计量资料以均数±标准差表示，率的比较采用*χ*^2^检验，均数比较采用两独立样本*t*检验，组间比较采用单因素方差分析。非正态分布的计量资料采用中位数（范围）表示，组间比较采用Wilcoxon秩和检验。采用单因素及多因素Logistic回归模型分析低钙血症的影响因素。*P*<0.05为差异有统计学意义。

## 结果

1. 基线资料：本研究共纳入80例患者，中位年龄59（41～90）岁，男49例（61％），遗传学高危35例（44％），包括1q21扩增25例、del（17p）8例、t（4；14）4例，含双打击2例[9]。病理性骨折41例（51％），骨折部位：脊柱27例，肋骨8例，脊柱+肋骨4例，胸骨1例，股骨颈1例。18例SRI患者（含2例透析）接受DENOS治疗；62例非SRI患者（CrCl≥30 ml/min）中30例接受DENOS治疗，32例接受ZOL治疗。80例MBD患者的基线特征见表1。

2. BTMs变化：40例3～4级MBD患者于诱导治疗期间接受了血清BTMs监测，包括DENOS组25例（SRI 10例，非SRI 15例）和ZOL组15例。10例SRI患者中8例接受VPd方案治疗，2例接受VCd方案治疗；30例非SRI患者接受VRd方案治疗。

各组基线平均骨吸收标志物β-CTX均高于正常，DENOS组SRI、非SRI患者较ZOL组升高更为显著（2.37 ng/ml对1.10 ng/ml对1.04 ng/ml，*P*＝0.035）。部分患者基线骨形成标志物PINP低于正常，亦有患者高于正常水平，但升高率低于β-CTX。抗骨吸收（AR）药物治疗后1个月，DENOS两亚组（SRI、非SRI）的血β-CTX降低率显著大于ZOL组（68％对59％对27％，*P*<0.001）（图1），说明DENOS抑制骨吸收的作用大于ZOL，SRI组降低率最高；各组PINP均升高，DENOS两亚组（SRI、非SRI）升高率亦大于ZOL组（34％对20％对11％，*P*＝0.043）（图2）。AR药物治疗1个月后各组iPTH较基线水平均升高，第2个月继续升高（DENOS组SRI患者177％，非SRI患者167％；ZOL组患者151％）后逐渐回落（图3）。

**图1:**
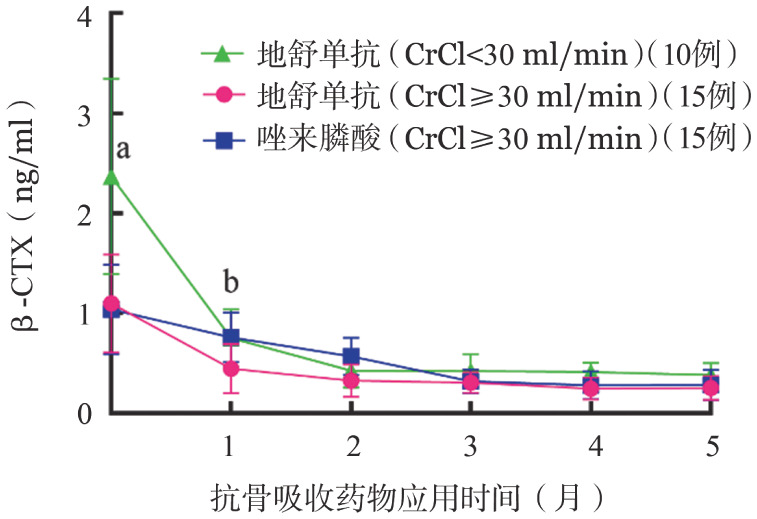
应用抗骨吸收药物后Ⅰ型胶原交联羧基端肽β特殊序列（β-CTX）变化 **注** CrCl：内生肌酐清除率；三组比较，^a^*P*<0.01，^b^*P*<0.05

**图2:**
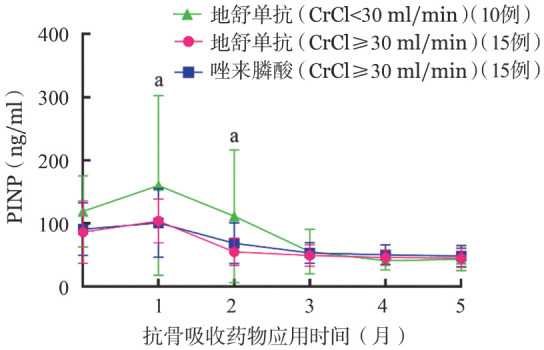
应用抗骨吸收药物后Ⅰ型原胶原氨基端前肽（PINP）变化 **注** CrCl：内生肌酐清除率；三组比较，^a^*P*<0.05

**图3:**
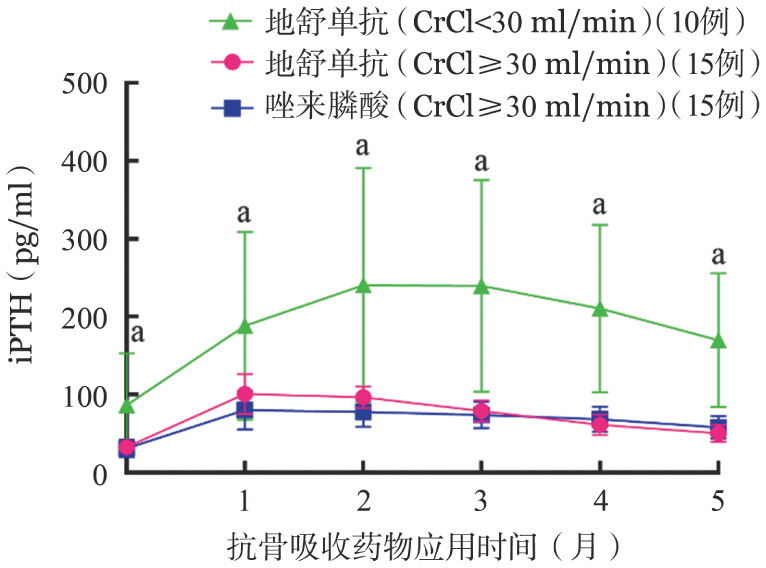
应用抗骨吸收药物后全段甲状旁腺激素（iPTH）变化 **注** CrCl：内生肌酐清除率；三组比较，^a^*P*<0.01

3. 血液学疗效与生存：接受DENOS治疗的SRI组、非SRI组和接受ZOL治疗组的中位随访时间分别为16（5～25）个月、18（6～26）个月和17（6～25）个月，至随访结束，三组血液学总有效率（77％对76％对80％）及CR/sCR率（40％对39％对42％）的差异均无统计学意义（*P*值均>0.05）。随访期间，16例进展，11例死亡，其中8例因疾病进展死亡，3例分别因冠状动脉性心脏病、肺炎、消化道出血死亡，因随访期较短，各组中位PFS及OS期均未达到。

4. 不良事件：26例（33％）患者发生低钙血症，22例发生于首次用药，4例于第2次用药时再次发生，最低校正血清钙为（1.86±0.14）mmol/L，2例SRI患者发生有症状低钙血症（腿部肌肉痉挛）。接受DENOS治疗的非SRI组患者低钙血症发生率高于ZOL组患者［20％（6/30）对13％（4/32），*P*＝0.028］，3级低钙血症发生率的差异无统计学意义［3.3％（1/30）对3.1％（1/32），*P*＝0.382］，两组的中位低钙血症发生时间分别为用药后第7（3～20）天和第10（4～25）天。SRI患者低钙血症发生率为89％（16/18），3级低钙血症发生率22％（4/18），中位低钙血症发生时间为治疗后第8（1～26）天，最低校正血清钙（1.81±0.11）mmol/L。12例基线时有高钙血症，治疗后9例发生低钙血症，包括DENOS组8例（SRI 6例，非SRI 2例）和ZOL组1例。

分析DENOS相关低钙血症的影响因素，单因素分析显示基线CrCl<30 ml/min［*OR*＝32.00（95％*CI* 5.73～178.85），*P*<0.001］及血钙≥2.75 mmol/L［*OR*＝14.29（95％*CI* 1.62～126.30），*P*＝0.017］与低钙血症发生相关，MBD≥3级、DS分期Ⅲ期与低钙血症的发生无关（*P*值均>0.05）。多因素分析显示，基线CrCl与低钙血症的发生相关［*OR*＝0.947（95％*CI* 0.920～0.975），*P*<0.001］。

随访期间入组患者均未发生AR药物相关颌骨坏死（ONJ）及肾功能损害，1例SRI患者因病情进展致血肌酐较前升高，SRI患者末次随访的中位CrCl水平较基线时升高162％（42 ml/min对16 ml/min，*P*＝0.014）。

## 讨论

MBD是MM的特征性表现之一，RANK/RANKL/OPG是关键通路，MM细胞恶性增殖，与骨髓基质细胞相互作用，释放破骨细胞（OC）活化因子，导致OC高度活化。过多分泌的RANKL与OC前体RANK受体结合，促进OC分化、成熟、激活[10]。DENOS作为RANKL抑制剂，阻断了该通路，抑制OC活性[11]。Raje等[3]的一项随机对照Ⅲ期研究显示，DENOS在延长首次SREs发生时间方面不劣于ZOL，而该研究未结合BTMs变化进行药效对比，且将CrCl<30 ml/min患者排除在外。DENOS治疗SRI-MM的Ⅱ期临床试验（NCT02833610）尚在进行中。本研究通过真实世界数据分析证实了DENOS治疗SRI的安全性，并结合BTMs变化证明DENOS抑制骨吸收的作用强于ZOL，并观察到低钙血症常发生于AR药物首次治疗后，DENOS组低钙血症发生率高于ZOL组，且SRI患者低钙血症发生率更高。

BPs是治疗MBD的经典药物，通过与骨矿物质结合抑制OC对骨表面的吸附，并被OC选择性吸收而抑制其活性。BPs以原形经肾脏排泄[12]，静脉给药的最初24 h，27％～62％药物与骨组织结合，此后被缓慢释放到循环系统经肾脏清除[13]。当药物剂量超过肾脏排泄负荷时可致肾损害，因此BPs禁用于CrCl<30 ml/min及急性肾损伤患者[14]。ZOL为第三代BPs，亦有药物相关急性肾小管坏死的报道[15]，基于其肾损害及骨组织内蓄积风险，ZOL用药方案多为4 mg，每4周1次，疗程2年。

DENOS是全人源化IgG2单克隆抗体，通过细胞内酶降解形式清除，包括靶点介导清除与网状内皮系统分解代谢，DENOS平均半衰期为26 d[16]–[17]。FREEDOM扩展研究的10年DENOS用药随访显示，不同程度肾功能损害（CrCl≥30 ml/min）者的不良事件、骨密度增加及骨折发生率相近，且长期暴露于DENOS未加重肾脏损害[18]。另一项研究中，55例（含8例透析）受试者均接受1次DENOS 60 mg治疗，结果显示其药代与药效动力学不受肾功能影响，且药物未被血液透析滤过，无需调整剂量[4]。本研究18例SRI（含2例透析）患者中位接受DENOS治疗16（5～22）次，未发生DENOS相关肾损害加重，药效未受影响，亦未调整剂量。

本研究AR药物治疗1个月后，DENOS组β-CTX降低率以及PINP升高率均大于ZOL组，此时各组骨代谢净效应为骨形成，DENOS组SRI患者尤为显著，而骨形成过程中钙离子回流可导致低钙血症，与本研究观察到的低钙血症发生特点相符。慢性肾功能不全可继发iPTH分泌过多[4]，以SRI且透析者最为显著，使得SRI组iPTH波动较大，统计分析时标准差较高。AR药物治疗后1个月各组iPTH较基线水平均升高，考虑为代偿性分泌以促进骨吸收，维持骨代谢平衡。

有学者发现肾功能不全者接受DENOS治疗后低钙血症发生率显著升高，且重度肾损害患者低钙血症的严重程度更高[4],[19]，有学者认为这一现象与慢性肾功能不全继发iPTH分泌过多导致高骨转换相关，iPTH使RANKL表达上调，骨吸收异常活跃，而与骨吸收相互制约的骨形成相对受抑，导致骨代谢失衡。由于首剂DENOS阻断了骨吸收，而骨形成在继续，此时如未充分补钙则易发生低钙血症[4]。一项正在进行的Ⅱ期研究（NCT02833610）采用DENOS治疗SRI患者，初期结果显示低钙血症发生率35％（7/20），受试者每日至少补充柠檬酸钙2 000 mg和维生素D 1 000 IU，此剂量显著高于将CrCl<30 ml/min患者排除在外的前瞻性研究[3],[20]。本研究SRI患者低钙血症率发生率（89％）显著增高，钙剂与维生素D摄取相对不足也是原因之一。

AR药物另一不良反应是ONJ, Raje等[3]报道的NDMM的Ⅲ期研究结果显示，DENOS与ZOL治疗后ONJ发生率的差异无统计学意义（4％对3％，*P*＝0.147），中位发生时间分别为17.3个月和13.6个月，DENOS组ONJ缓解率更高，与其药物机制有关。本研究入组患者随访期间未发生ONJ，入组病例少是原因之一，保持良好的口腔卫生及口腔科操作前后严格遵守AR药物停用原则可降低ONJ发生风险。

总之，MBD的理想治疗模式是有效的抗骨髓瘤治疗联合AR药物，能够在降低肿瘤负荷的同时恢复正常骨代谢。DENOS的疗效、安全性、便捷性均具有一定优势，为MBD的标准化治疗带来新的选择。低钙血症是DENOS的常见不良反应，但一般为轻中度且可控，充分补钙及维生素D可降低低钙血症的发生风险。
